# Genetic diversity of *Plasmodium falciparum* and genetic profile in children affected by uncomplicated malaria in Cameroon

**DOI:** 10.1186/s12936-020-03161-4

**Published:** 2020-03-18

**Authors:** Theresia Njuabe Metoh, Jun-Hu Chen, Philip Fon-Gah, Xia Zhou, Roger Moyou-Somo, Xiao-Nong Zhou

**Affiliations:** 1 Department of Biochemistry, Faculty of Science, The University of Bamenda, P. O. Box 39 Bambili, Bamenda, Cameroon; 2 National Institute of Parasitic Diseases, Chinese Centre for Disease Control and Prevention, Shanghai, 200025 People’s Republic of China; 3 WHO Collaborating Centre for Malaria, Schistosomiasis and Filariasis, Key Laboratory of Parasite and Vector Biology, Ministry of Health, Shanghai, 200025 People’s Republic of China; 4 ITC Enschede, University of Twenty, Hengelosestraat 99, 7514 AE Enschede, The Netherlands; 5 Department of Geoscience-Remote Sensing and GIS, The University of Bamenda, P. O. Box 39 Bambili, Bamenda, Cameroon; 6 Institute of Medical Research and Medicinal Plants-IMPM, P. O. 6123, Yaoundé, Cameroon; 7 Faculty of Medicines and Biomedical Sciences, The University of Yaoundé I, P. O. Box 812, Yaoundé, Cameroon

**Keywords:** MSP-1, MSP-2, GLURP protein, Plasmodium, Heterozygote, Infection control

## Abstract

**Background:**

Malaria is a major public health problem in Cameroon. The study of the genetic diversity within parasite population is essential for understanding the mechanism underlying malaria pathology and to determine parasite clones profile in an infection, for proper malaria control strategies. The objective of this study was to perform a molecular characterization of highly polymorphic genetic markers of *Plasmodium falciparum*, and to determine allelic distribution with their influencing factors valuable to investigate malaria transmission dynamics in Cameroon.

**Methods:**

A total of 350 *P. falciparum* clinical isolates were characterized by genotyping block 2 of *msp*-*1*, block 3 of *msp*-*2*, and region II of *glurp* gene using nested PCR and DNA sequencing between 2012 and 2013.

**Results:**

A total of 5 different genotypes with fragment sizes ranging from 597 to 817 bp were recorded for GLURP. Overall, 16 MSP-1 genotypes, including K1, MAD20 and RO33 were identified, ranging from 153 to 335 bp. A peculiarity about this study is the RO33 monomorphic pattern revealed among the *Pfmsp*-*1* allelic type. Again, this study identified 27 different *Pfmsp*-*2* genotypes, ranging from 140 to 568 bp in size, including 15 belonging to the 3D7-type and 12 to the FC27 allelic families. The analysis of the MSP-1 and MSP-2 peptides indicates that the region of the alignment corresponding K1 polymorphism had the highest similarity in the MSP1and MSP2 clade followed by MAD20 with 93% to 100% homology. Therefore, population structure of *P. falciparum* isolates is identical to that of other areas in Africa, suggesting that vaccine developed with K1 and MAD20 of *Pfmsp1* allelic variant could be protective for Africa children but these findings requires further genetic and immunological investigations. The multiplicity of infection (MOI) was significantly higher (P < 0.05) for *Pfmsp*-*2* loci (3.82), as compare with *Pfmsp*-*1* (2.51) and heterozygotes ranged from 0.55 for *Pfmsp*-*1* to 0.96 for *Pfmsp*-*2*.

**Conclusion:**

High genetic diversity and allelic frequencies in *P. falciparum* isolates indicate a persisting high level of transmission. This study advocate for an intensification of the malaria control strategies in Cameroon.

*Trial registration* This study was approved by Cameroon National Ethics Committee. It is a randomized controlled trial retrospectively registered in NIH U.S. National Library of Medicine, ClinicalTrials.gov on the 28/11/2016 at https://clinicaltrials.gov/ct2/show/NCT02974348 with the registration number NCT02974348

## Background

In spite of enhanced control efforts, malaria continues to be a major public health problem in sub-Saharan Africa and south-eastern Asia, and *Plasmodium falciparum* infection is prevalent in most of the endemic countries. The World Health Organization (WHO) estimated that 219 million malaria cases occurred worldwide of which 78% were in Africa [1]. About 407,000 fatal cases were registered, 92% in Africa and 61% of the global death in children under 5 years of age, with *P. falciparum* being the major cause of all deaths [1, 2]. Malaria in Cameroon is caused by three human malaria species: *P. falciparum, Plasmodium ovale* and *Plasmodium malariae.* The prevalence of *P. falciparum* is about 99% while *P. malariae* and *P. ovale* share the remaining 1%. *Anopheles arabiensis*, a member of the *Anopheles gambiae* complex, is a principal malaria vector in Cameroon [3]. Malaria is a leading public health problem in Cameroon and is reported as the first cause of morbidity and mortality accounting for 16% outpatient visits, 20% hospital admissions and 27% inpatient deaths [4].

Despite the current efforts to control malaria in Cameroon, the situation has not improved, mainly due to the increasing vector resistance to insecticides [5], and resistance to almost all anti-malarial drugs, including some resistance to artemisinin-based combination therapy (ACT) [6, 7]. Merozoite surface protein (MSP-1) and merozoite surface protein-2 (MSP-2) are two proteins challenging the human immune system [8] and are important candidates for development of blood stage malaria vaccines [9]. The *msp*-*1* gene is located on chromosome 9 and contains 17 blocks of sequences, of which 7 are variable, flanked by conserved regions [10]. The block 2 *msp*-*1* is particularly polymorphic and 3 distinct allelic families have been described as MAD20, K1 and RO33. The *msp*-*2* gene is located on chromosome 2 composed of five blocks of which the most polymorphic is the central block 3.

The polymorphic central domain of the gene encoding MSP-2 belongs to 2 distinct families; 3D7 and Fc27 [11, 12]. Allelic forms of these antigen genes have been reported from different parts of the world [12–14]. Differences in allelic types as well as the number of repeat sequences in *msp*-*1* and *msp*-*2* can be detected by PCR, followed by dot-blot hybridization [15]. Size polymorphism is used not only for the *msp* genes but also for other markers genes, including glutamate rich protein (*glurp*) and circumsporozoite protein (*csp)* in which variations are detected by sequencing. Genetic diversity of *P. falciparum* populations determines the intensity of malaria transmission [16, 17], thereby providing a baseline data for any anti-malarial drug efficacy trials and the possibility of implementing control strategies based on modern interventions or vaccines. Merozoite surface proteins 1 and 2 genotyping is widely used in malaria molecular epidemiology studies to assess the allelic diversity and multiplicity of infection as a proxy of transmission level in molecular monitoring of interventions [18]. The *Pfmsp1*, *Pfmsp2* and *glurp* markers are commonly selected because they are located on different chromosomes, and this reduces the likelihood of a linkage [19, 20]. Genotyping of these genes has been effectively used to trace individual clones over time in cohort studies and to measure duration of infection [21].

These markers are equally essential in distinguishing recrudescence from reinfection of the parasite in an anti-malarial treatment trial [22], since the discriminating power of these markers is dependent on the extent of allelic diversity and on the frequency of each allele within a population [12]. Genetic diversity of *P. falciparum* has been used to implement specific strategies for control and to evaluate the impact of interventions on changes in malaria epidemiology [5, 23]. However, in Cameroon, there are no data on the multiplicity of infection (MOI) I and limited data on *P. falciparum* genetic diversity, especially when *msp*-*1, msp*-*2* and *glurp* need to be considered together as molecular markers in genotyping studies.

Thus, the aim of this study was to characterize the highly polymorphic genetic markers of *P. falciparum* field isolates, including the merozoite surface protein 1 (MSP-1), the merozoite surface protein 2 (MSP-2) and glutamate rich protein (GLURP), and to determine the allele distribution and factors influencing the MOI and heterozygosity as indicators of malaria parasite transmission dynamics.

## Methods

### Study site

**Fig. 1:**
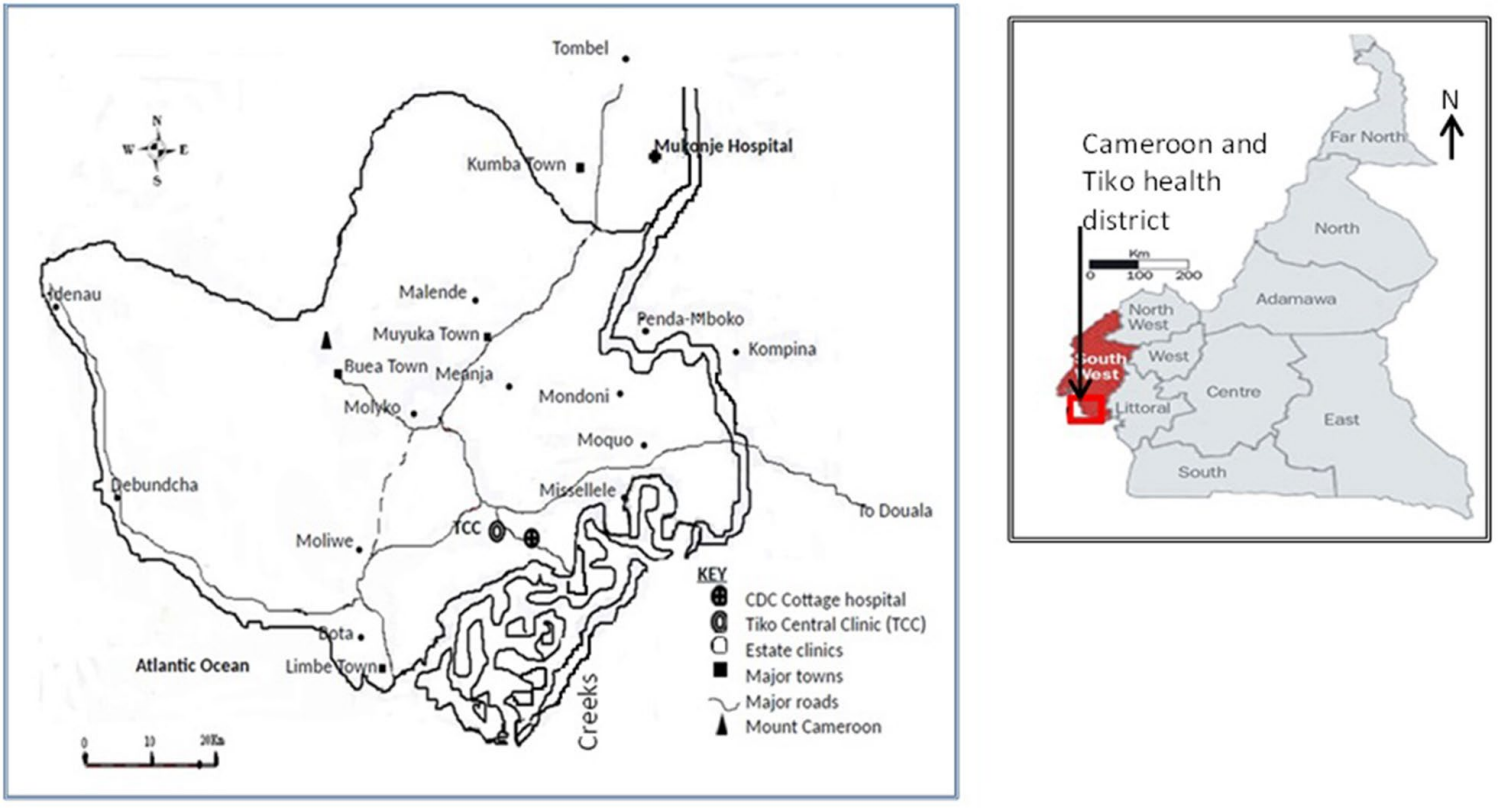
Map of major health facilities of the Cameroon Development Corporation (CDC), South West Region of Cameroon and Geographic map of Cameroon. Source: https://www.worldatlas.com/webimage/countrys/africa/cm.htm

This study was carried out in the areas occupied by the Cameroon Development Corporation (CDC). This is an agro-industrial para-state company practicing plantation farming based in the south-west region of Cameroon, specialized in the production of rubber, palm oil, and banana. Cameroon climate which varies with terrain, from equatorial rain forest with mean annual temperature of 24.5 °C in the south coastal regions to 26.5 °C in the semiarid northern regions. Elevation extremes are 0 m at sea level at the Atlantic Ocean in the south west and with highest peak at Mount Fako (Mount Cameroon) at 4095 m above the sea level. The CDC is rural communities in the South West region of the country located at 45 km away from Douala (Fig. 1). The climate is characterized by fairly constant temperatures and two seasons: a short dry season (November–February) and a long rainy season (March–November) with abundant precipitation (2000–10,000 mm). The mean annual rainfall is 2625 mm, relative humidity is constantly high (75–80%), and the temperature varies from 18 °C in August to 24.5 °C in March [3]. The CDC Company has headquarters in Limbe town, with a multicultural population of about 45,000 plantation workers consisting primarily of peasant farmers, with over 60% coming from the north-west and western regions of Cameroon. Limbe has 8 primary and two secondary schools. The community has good access roads, good pipe-borne water and electricity supplies in some localities. CDC has good health system consisting of two reference hospitals, Tiko and cottage hospital which are primary health facilities belonging to the Tiko health District and, 23 satellite clinics located in different CDC estates of the south west region of Cameroon. Apart from the CDC Primary Health Centre and hospitals, there are also private clinics, mission and government health centres and hospitals. This site was selected to conduct both clinical trial studies with artemisinin-based combination therapy and malaria parasite genetic diversity studies.

The study site is harbouring all *Plasmodium* species found in the country. Malaria transmission in this area is perennial with high intensity and prevalence varying from 7 to 29% [24] with peak periods corresponding to the rainy season. Three *Anopheles* vectors including *An. gambiae* sensu lato, *Anopheles funestus* and *Anopheles nili* have been identified in malaria transmission in the South West Region. *Anopheles gambiae*, the most dominant in terms of aggressiveness and activity, accounts for up to 72.7% of transmission, Infection rates of 87 infective bites/person/year [3] and overall entomologic inoculation rate (EIR) estimated recently at 3.93 infective bites/person/night [24]. In addition, houses are grouped into camps which are structured buildings provided by the company to accommodate the workers and their families. Because the patients coming to CDC health facilities are located in various camps and grouped in well-organized communities it makes it easy for the investigators to track them after enrolment at D0, in case they do not return for follow-up at health facilities. This has an impact in reducing the rate of loss for patients follow-up. Besides, this study site is one of the settings carefully selected by the NMCP as sentinel site to carry out epidemiological survey in order to obtain information paramount in guiding the national malaria management policy.

### Sampling and parasite collection

The sample size was estimated using the following formula for sample size calculation as previously described [25]. n = Z^2^ * p (1 − p)/e^2^, Z = 1.96, p = prevalence of malaria in Cameroon (29%), e = error rate = 0.05; n = 317 patients, with 10% increment in case of PCR failure, making a total of 350 patients. The *P. falciparum* isolates were collected from microscopically diagnosed *P. falciparum* positive patients aged 6 months to 6 years, in clinics and hospitals of CDC Cameroon. Blood sample was taken from each patient using finger prick for thick and thin blood films. Two to three drops of blood were collected on 3 MM Whatman filter paper. A total of 350 blood samples were collected from 23 clinics and 2 hospitals of the CDC Cameroon belonging to 2 different health districts.

### Parasitaemia estimation

Blood films were stained with 10% Giemsa at pH7.2 and then examined microscopically for the presence of malaria parasites; 200 fields under 1000× magnification were examined from the thick film before the slide was considered negative. For positive slides, parasitaemia (parasite density) was determined by counting only the asexual stages against 200 white blood cells (WBC) and then multiplied by 25, assuming the average of total WBC count of individuals equal to 8000 cells/μl of blood [26, 27]. The level of parasitaemia was graded as low (< 1000 parasites/μl of blood), moderate (1000–9999 parasites/μl of blood) and severe (≥ 10,000 parasites/μl of blood) [28].

### Genomic DNA extraction and genotyping

Genomic DNA was extracted from blood spots collected on filter papers. Briefly, a disc of the filter paper was punched out from the blood spot using a paper puncher and placed in 1.5 ml centrifuge tubes using clean forceps. Genomic DNA was extracted using Qiagen blood and tissue kit (QIAGEN, Germany) according to the manufacturer’s instructions.

### PCR amplification and allele detection

DNA was eluted using 50 μl AE (10 mM Tris–Cl; 0.5 mM EDTA; pH 9.0) elution buffer (QIAGEN, DNeasy^®^ Blood & Tissue Kit, Cat. no. 69506, Germany) and kept at − 20 °C until used for PCR. Genomic DNA was amplified by primary and Nested PCR using allelic specific Primers (Table 1) and conditions for PCR amplification were followed as previously described [8, 19] for family specific allele analysis of *msp*-1 (block 2), *msp*-2 (block 3) and *glurp* (region II). In the primary PCR, a 25 μl PCR mixture was used containing 1 μl of DNA template, 0.2 µM of each primer, 1× TBE buffer, 250 μM of dNTPs, nuclease free water, 1 U of Taq polymerase enzyme, 2 mM MgCl2, 10 mM KCl, and 10 mM Tris–HCl, at pH 8.3. All reagents were from TIAGEN (Biotechnology, Inc., Beijing). Cycling conditions for the primary PCR were as follows; starting with three single steps of denaturation at 94 °C for 5 min, annealing at 58 °C for 2 min and extension at 72 °C for 2 min. This was followed by 35 cycles of denaturation at 94 °C for 1 min, annealing at 58 °C for 1 min and extension at 72 °C for 1 min, then a single annealing step at 58 °C for 2 min and final extension at 72 °C for 10 min. *P. falciparum* genotypes were further analysed by amplification of the two highly polymorphic regions of *msp*-*1* (Block 2) and *msp*-*2* (Block 3) using nested-PCR as previously described [19] with slight modifications for the cycling conditions of the secondary PCR. Briefly, oligonucleotide primers sets (Table 1), were used for detecting the family-specific (K1, MAD20 and RO33 in MSP-1; FC27 and IC in MSP-2). Three microlitres of primary PCR products were used as the DNA templates in the secondary PCR, which had similar concentrations to the primary PCR. The cycling conditions for the secondary PCR were as follows: starting with a single step of denaturation at 95 °C for 10 min followed by 35 cycles of denaturation at 94 °C for 30 s, annealing at 58 °C for 30 s and extension at 72 °C for 1 min, and a final extension at 72 °C for 10 min. PCR reaction mixtures were incubated in a thermal cycler Perkin-Elmer Cetus PE 9600 (*Norwalk*, *CT)*. The secondary PCR products were separated by electrophoresis at 100 V on 1.5% molecular grade agarose gel (Caisson, Utah, USA), stained with ethidium bromide, submerged in 0.5 × TBE (Tris–borate EDTA) buffer and visualized by UV transilluminator (BioDoc-It UVP, Cambridge, UK) at 302 nm on gel documentation system. The number and size of DNA fragments were estimated based on their mobility related to a 100 bp DNA ladder (Vivantis, Selangor Darul Ehsan, Malaysia). DNA fragment sizes were binned into different classes of 20 and 50 bp ranges with each bin assigned as an allele. Alleles in paired samples were considered a match for *msp2* and *msp1* if within 20 base pairs and for *glurp* if within 50 base pairs [11, 14].

**Table 1:** Sequences of primers for *msp1*, *msp2* and *glurp* genes of *P. falciparum* from Cameroon

*Msp*-*1* (block 2)	
Primary PCR	CHM1-OF: 5′ CTAGAAGCTTTAGAAGATGCAGTATTG-3′ CHM1-OR: 5′ CTTAAATAGTATTCTAATTCAAGTGGATCA-3′
Secondary PCR	CHM1-KF: 5′ AAATGAAGAAGAAATTACTACAAAAGGTGC-3′ CHM1-KR: 5′ GCTTGCATCAGCTGGAGGGCTTGCACCAGA-3′ CHM1-MF: 5′ AAATGAAGGAACAAGTGGAACAGCTGTTAC-3′ CHM1-MR: 5′ ATCTGAAGGATTTGTACGTCTTGAATTACC-3′ CHM1-RF: 5′TAAAGGATGGAGCAAATACTCAAGTTGTTG-3′ CHM1-RR: 5′ CATTTGAAGGATTTGCAGCACCTGGAGATC-3′
*Msp*-*2* (block 3)	
Primary PCR	M2-OF: 5′ ATGAAGGTAATTAAAACATTGTCTATTATA-3′ M2-OR: 5′ CTTTGTTACCATCGGTACATTCTT’3′
Secondary PCR	M2-FCF: 5′ ATATTAAGAGTGTAGGTGCARATGCTCCA-3′ M2-FCR: 5′ TTTTATTTGGTGCATTGCCAGAACTTGAAC-3′ M2-ICF: 5′AGAAGTATGGCAGAAAGTAAKCCTYCTACT-3′ M2-ICR: 5′ GATTGTAATTCGGGGGATTCAGTTTGTTCG-3′
*Glurp* (region II)	
Primary PCR	CHG-OF: 5′ -TGAATTTGAAGATGTTCACACTGAAC-3′ 3′ CHG-OR: 5′ -GTG GAATTGCTTTTTCTTCAACACTAA-3′
Secondary PCR	CHG-NF: 5′-TGAATTTGA AGA TGT TCA CAC TGA AC-3′ CHG-OR: 5′-GTG GAATTGCTT TTTCTTCAACAC TAA-3′

### Multiplicity of infection (MOI) and expected heterozygosity (HE)

Multiclonal infections were defined as those having more than one allele in at least one locus out of the loci genotyped. The MOI was determined by calculating the number of different alleles at any one locus detected in the sample [9]; single infections were those with only one allele per locus at all of the genotyped loci. The mean MOI was determined as the quotient of the total number of *P. falciparum* genotypes detected in MSP-1 or MSP-2 by the number of samples positive for either *msp*-*1* or *msp*-*2* [29].

As a measure for genetic diversity, the expected heterozygosity (HE) which represents the probability of being infected by two parasites with different alleles at a given locus and ranging between 0 and 1, [12] was calculated by using the following formula:

$$ {\text{HE}}\, = \,\left[ {{\text{n}}/\left( {{\text{n}} - 1} \right)} \right] \, [( 1- \varSigma {\text{pi}}^{ 2} )], $$

where n is the number of isolates sampled and pi is the allele frequency at a given locus [29].

### Statistical analysis

Data was analysed using the SPSS for windows software version 17 of the Statistical Package of Social Science (SPSS) (SPSS Inc, Chicago, IL, USA) [30]. For descriptive analysis, proportion was used to present the distribution of different allelic families while the mean was used to present the MOI. Independent. Independent one way ANOVA method was used to assess the relative size of variance among group means (between group variance) compared to the average variance within groups (within group variance). Thus this method was used to compare the mean MOI according to, gender, parasitaemia and across *msp1*, *msp2* and *glurp* family specific alleles. The F value calculated from the observed data with the critical value set at an α error level of 0.05 in the F table. A P-value ≤ 0.05 was considered indicative of a statistically significant difference.

### *msp*-*1* and *msp*-*2* sequence analysis

Purified PCR products of isolates representing different alleles of *msp*-*1* and *msp*-*2* were sequenced in both directions with the primers of the secondary PCR using the ABI PRISM^®^ BigDyeTM terminator Ready Reaction Cycle Sequencing Kit (Biometra Thermocycler, England) according to the manufacturer’s instruction. The sequences were then analysed using the DNASTAR software package (DNASTAR, Madison, WI). The sequences were used to correct the estimated molecular weight and to confirm the nature and size of the amplified product. To understand the identity of Cameroonian isolates with respect to isolates of other regions, sequence data available in public domains were downloaded for allelic families of *msp*-*1* and *msp*-*2* and aligned using ClustalW method (EMBL-EBI, Hixton, and Cambridge, UK), with query sequences and word match set at 50 and 99% as identity threshold, in a FASTA file database then analysed by MEGA version 5.1 [31]. Sequence differences were identified using BLASTN sequence homology searches. Each allelic family *msp*-*1* (including K1, MAD20 and R033) and *msp*-*2* (including FC27 and 3D7/IC) were analysed separately in order to estimate the average number of nucleotide substitutions for each *msp*-*2* allele, and to examine the mode of evolution of FC27 type repeats. Individual repeat units were assembled and aligned with DNAstar and BLAST searched in GenBank to analyse the distribution of the pairwise proportion of nucleotide differences among individual repeat units using isolates derived from GenBank, namely the isolate from Gabon accession number AY372506 for FC27, the isolate HM568631 from India for 3D7/IC allelic family. As for *msp*-*1* alleles, the isolate from Brazil (accession:JX416338) for the alignment of K1-type allele, while the isolates EU032224 from central sub-Saharan Africa and AY138508 from Iran were used as reference isolate for the sequence analysis of R033 and MAD20, respectively [32].

### Ethical approval and consent to participate

This study was approved by the scientific and Ethical Committee of the Cameroon Ministry of Public Health. Field administrative approval was provided by the health administrative authorities of the CDC. A written informed consent for voluntary participation in this study was obtained from parents or guardians of children before their enrolment in this study.

## Results

### Study profile

Out of 315 DNA samples at baseline, 137 samples for *msp*-*1* and 307 samples for *msp*-*2* were successfully amplified by PCR, among which 107 for *msp*-*1* and 243 for *msp*-*2* were successfully sequenced.

### Genetic diversity and haplotype frequency

The number of genotypes observed at each marker is shown in Table 2. Sixteen (16) different *msp*-*1* genotypes were observed, representing K1 (9 genotypes), MAD20 (6 genotypes) and RO33 (1 genotype) allelic families. The *msp*-*1* fragment sizes ranged from 153–335 bp, while the *msp*-*2* fragments ranged from 140 to 568 bp both for the FC27 and IC allelic families (Table 2). The majority (87.5%) of these genotypes especially for the k1 (100%) and MAD20 (97.5%) belonging to the *msp1* allelic family occurred at a frequency below 10% (Table 2). However, one genotype of the MAD20 family (167–187 bp) occurred above 10%. The R033 family was found to be monomorphic with an amplified fragment size of 155 bp and occurred at a frequency of 78.1% (107/137) (Tables 2 and 4) of the overall *msp1* genotypes.

**Table 2:** Base pair range and number of detected genotypes of the respective *msp*-*1* and *msp*-*2* gene families in *P. falciparum* isolates from 315 malaria patients in Cameroon

Marker	*msp*-*1*			*msp*-*2*	
	K1	MAD20	R033	FC27	3D7/IC
Number of different genotypes per allele	9	6	1	12	15
Allele range (bp)	153–335	175–205	155	140–387	200–568
Total per locus	16			27	
Genotypes frequency for allele type occurring at a frequency < 10%	9 (100%)	5 (97.5%)	0 (0%)	10 (83.3%)	14 (95.3%)
Total per allele	14 (87.5%)			24 (88.9%)	
Genotypes frequency for allele type occurring at a frequency > 10%	0 (0%)	1 (16.7%)	1 (100%)	2 (16.7%)	1 (6.7%)
Total per allele	2 (12.5%)			3 (11.1%)	

A total of 27 different *msp*-*2* genotypes (size range from 140 to 568 bp) were recorded of which 15 belonged to the 3D7-type and 12 to the FC27 allelic families (Table 2). The majority of these genotypes occurred at a frequency below 10%. However, 1 genotype from the 3D7/IC allelic family (420–440 bp), and 2 from the FC27 family (176–201 bp and 201–226 bp) occurred above 10%. The 3D7/IC family was found to be highly polymorphic (P = 0.01) as compared to FC27.

The *glurp* diversity showed 5 different *glurp* genotypes (size range from 597 to 817 bp) with the majority of the allelic families occurring at above 10% (Table 3). However, only two alleles occurred below 10% of which, 4.67% and 9.34% belonged to the 550–600 bp and 652–702 bp allelic group.

**Table 3:** Distribution of allelic variants of GLURP RII repeat region of *P. falciparum* isolates amongst malaria patients in Cameroon

Genotypes	Allelic size variants (bp)	Frequency (%)	No. fragments	MOI
I	550–600	5 (4.5)	5	1.00
II	652–702	18 (16.36)	19	1.10
III	754–804	63 (57.3)	64	1.10
IV	805–855	14 (12.7)	14	1.00
V	856–906	10 (9.1)	10	1.00
Total glurp		110	112	1.02

**Fig. 2:**
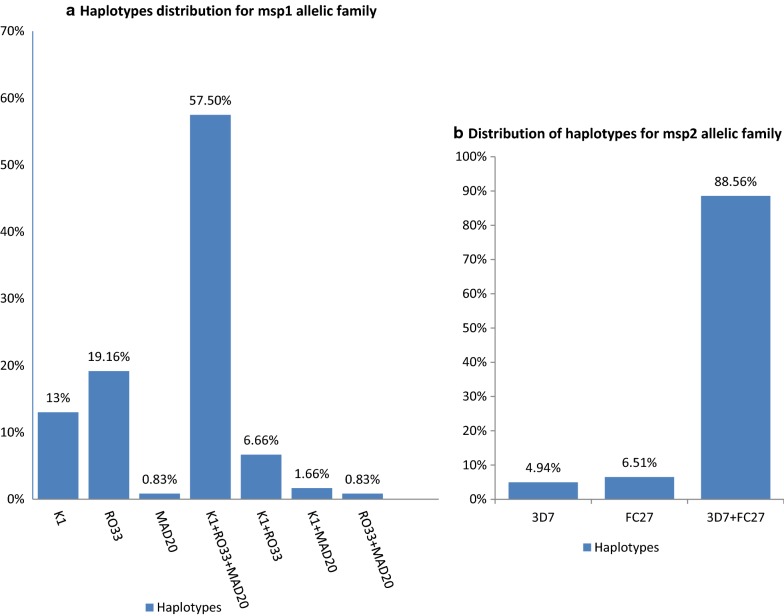
**a** Distribution of *msp*-*1 haplotypes.***b** Distribution of haplotypes for msp2 allelic family

This study revealed that more than half of the *msp*-*1* positive samples harboured all the three types of alleles of the *msp*-*1* gene. Thus the combination of RO33, MAD20 and K1 allelic families was identified with an overall frequency of 57.50% (Fig. 2a). The RO33 allelic family was predominant as it was identified in 78.10% (107/137) of the samples. One-third (33%) of the blood samples positive for *msp*-*1* were identified as monoclonal infections while two-third (66%) exhibited a polyclonal pattern of K1, MAD20 and R033 combination in a set of two or three alleles (Fig. 2a). Among the polyclonal infections, K1/RO33, K1/MAD20 and Ro33/MAD20 constituted 6.66%, 1.6% and 0.83% of the *msp*-*1* positive isolates respectively. The distribution of the identified *msp*-*2* allelic families is illustrated in Fig. 2b. Overall, 11.42% of *msp*-*2* positive isolates were identified as monoclonal infections either for 3D7/IC or FC27 allelic families against 88.56% *msp*-*2* positive isolates (Fig. 2b) displaying a polyclonal pattern of infections. The frequency of the 3D7/IC and FC27 haplotypes combinations was found to be higher than the frequency of samples with only 3D7 or FC27 allelic families (Fig. 2b).

### Multiplicity of infection and expected heterozygosity

*Plasmodium falciparum* isolates in this study had higher rates of multiple genotypes infection with an overall mean multiplicity of infection of 3.16. The mean MOI for *msp*-*2* loci was the highest (3.82), as compared with *msp*-*1* (2.51) (Table 4). In the children, there was no difference in mean MOI between male and female (P = 0.1) (Table 5). However, the influence of parasite density on genetic diversity showed that children with parasite density ranging from 2000 to 5000 had the lowest MOI (Table 6). Meanwhile those with parasite ranging from 20,000 and above exhibited the highest MOI difference (P = 0.001), as compared with various range of parasite densities. The overall mean multiplicity of infection, 3.16 observed for msp1 and msp2 is significantly higher (P < 0.05) than revealed among *glurp* genotypes (1.02) (Table 7).

**Table 4:** Heterozygosity, and multiplicity of infection (MOI) per msp1 and msp2 allelic families in *P. falciparum* isolates from 315 malaria patients in Cameroon

Family specific alleles	Frequency (%)	No. fragments	He	MOI
K1	80 (58.39)	197	0.65	2.46
MAD20	40 (29.19)	40	0.50	1.00
R033	107 (78.10)	107	0.50	1.00
Total/msp1 (N = 137)	227	344	0.55	2.51
3D7 (IC)	295 (96.1)	621	0.99	2.10
FC 27	298 (97.1)	552	0.94	1.88
Total/msp2 (N = 307)	593	1173	0.96	3.82

**Table 5:** Distribution of MOI by gender amongst Cameroonian children with falciparum malaria

	PCR+	No. 3D7/IC fragments	No. FC27 fragment	No. FC27 + 3D7 fragment	MOI	P-value
Female	157	43	21	175	1.52	0.08
Male	150	76	22	202	2	
Total	307	119	43	377		

F-ratio value = 3.7, df = 1; P = 0.08

**Table 6:** Distribution of msp2 allele by parasite density among Cameroonian children with falciparum malaria

Parasite count	PCR+	No. 3D7/IC fragments	No. FC27 fragments	No. FC27 + 3D7/IC fragments	Total fragments	MOI
2000–5000	123	54	39	95	188	1.53
5001–10,000	36	6	4	50	60	1.67
10,001–15,000	43	31	24	63	118	2.7
15,001–20,000	36	24	27	35	86	2.38
20,001–above	69	64	51	115	230	3.3
Total	307	179	145	358	682	2.2

**Table 7:** Distribution of MOI and HE across the three markers in Cameroonian isolates

Family specific alleles	N	genotypes	No. fragments	HE	MOI	P-value
Msp1	137	16	344	0.96	2.51	0.02
Msp2	307	27	1173	0.55	3.82	
Glurp	110	5	112	0.01	1.02	

F-ration value = 7.9; df = 1; P = 0.02

The mean MOI was found to be higher in male patients whose parasitaemia ranged from moderate to high level, than that of male patients with low parasitaemia. The expected heterozygosity (HE) was calculated which used to estimate the fraction of all parasites that would be heterozygous for any of the two loci. Table 4 shows generally high HE values in each allelic family ranging from 0.5 to 0.65 for *msp*-*1* and from 0.94 to 0.96 for *msp*-*2*.

### Sequence analysis of genetic polymorphism of *msp*-*1* block 2 and *msp*-*2* block3

The *msp*-*1* block 2 and *msp*-*2* block 3 DNA fragments from PCR products with either similar or different molecular sizes in agarose gels were sequenced with the aim of further estimating the genetic diversity of the parasite population. Partial gene sequences were obtained from each end of the PCR product using the same primers for the nested PCR reactions, then DNA sequences assembled and BLAST searched in GenBank to identify identical or similar sequences. Analysis of *msp*-*1* and *msp*-*2* sequence data revealed above 80% identity of study isolates among themselves in general and above 83% with isolates from other countries with a few exceptions (Table 8). Moreover, the RO33 allelic sequences of Cameroonian isolates were all identical and had shown above 99% similarity (Table 8) with sequences reported for isolates of Kenya [33]. In the K1 family, 93 to 99% similarity was observed with K1 allelic sequences reported for isolates of Tanzania [34], Indonesia (accession no. U72950), Vietnam and Brazil [35]. Likewise, 83 to 100% identity was observed in MAD20 family with isolates of Iran [36], Sudan [37], Vietnam [35]. Allelic families of *msp*-*2*, FC27 and 3D7/IC showed 97 to 100% identity with isolates of Gambia [38], Brazil (accession no. DQ115969), Tanzania (accession no. AY378316), and Gabon (accession no. AY372518). No significant identity was found with either the Nigerian or the Brazilian isolates with respect to the 3D7/IC allelic family of this study. However 89 to 97% identity were recorded with isolates from Gambia and Thailand [38].

**Table 8:** *Plasmodium falciparum msp*1 and *msp*2 haplotype sequence diversity amongst malaria patients in Cameroon compared with isolate from different geographical regions

Family specific allele	Nr of different genotypes	Actual fragment size range (bp)	Degree of identity between alleles of study isolates (%)	Reported identical sequences/accession	Degree of identity (%)	Origin	References
K1	9	153–335	90–99	AF509714	90–94	Brazil	Ferreira et al. [35]
				AF191061	93–98	Indonesia	Unpublished
				AF061134	93–99	Tanzania	Jiang et al. [68]
				AF509651	93–99	Vietnam	Ferreira et al. [35]
				M77730	93–99	Thailand	Jongwutiwes et al. [69]
				JX416338	99	Brazil	Unpublished
MAD20	5	175–205	83–92	AY714585	92	Brazil	Scopel et al. [70]
				AF251345	83–93	China	Unpublished
				AY138509	82	Iran	Unpublished
				AF034635	83	Sudan	Cavanagh et al. [37]
				AF509653	91	Vietnam	Ferreira et al. [35]
				M77722	83	Thailand	Jongwutiwes et al. [69]
				EU32224	100	Central Sub Saharan Africa	Noranate et al. [32]
R033	1	155	99–100	AY538507	99	Iran	Unpublished
				AF462453	99	Kenya	Takala et al. [33]
				AF191064	98	Indonesia	Unpublished
				M77737	99	Thailand	Jongwutiwes et al. [69]
				AY138508	98	Iran	Unpublished
IC	15	200–568	97	HM568631	97	India	Unpublished
FC27	12	140–387	97	DQ115973	97	Brazil	Peyerl-Hoffmann et al. [46]
				DQ338451	98	Iran	Unpublished
				AF329577	97	Ghana	Unpublished
				AF329579	96	PNG	Unpublished
				U91668	97	Gambia	Unpublished
				AY532386	98	Tanzania	Unpublished
				AF104696	99	Vietnam	Weisman et al. [71]
				AY372506	100	Gabon	Mayengue et al. [72]

## Discussion

The aim of this study was to perform a molecular characterization of highly polymorphic markers in *P. falciparum* clinical isolates. In this first study, conducted after the introduction of artemisinin-based combination therapy (ACT), it was observed that *P. falciparum* field isolates in Cameroon exhibited a high degree of genetic polymorphism in *P. falciparum msp*-*1* and *msp*-*2* markers. These genes encoding for individual functional proteins expressed on the surface of the merozoite appear to play an essential role in the invasion of the red blood cell [39] and are of interest as potential vaccine candidates and as drug targets for inhibiting blood-stage replication [40]. This study identified a high proportion of multiclonal isolates and MOI and a total of 16 different *Pfmsp*-1 and 27 different *Pfmsp*-*2* gene types. This figure is not exhaustive since it is obvious that with nested PCR genotyping some of the sub-populations present in mixed infections would not be fully typed by all amplification steps [21]. Moreover, more amplified product was found for the *msp2* gene than *msp1*, this could be explained by non-synonymous substitutions introduced in template DNA that could jeopardize the proper annealing of the primer at its binding site in *msp1* gene or could be explained by the fact that natural selection is more efficient when acting on *msp*-*1* than *msp*-*2* [39, 41]. This suggests that MSP-1 as compared with MSP-2 proteins are under strong functional constraints in a complex interaction with the host leading to an increased in host’s immunological response. The two markers including *msp*-*1* (16 genotypes) and *msp*-*2* (27 genotypes) revealed considerably greater parasite diversity than *glurp* (5 genotypes). Thus, the observed genetic polymorphism in the these two *P. falciparum* major merozoite surface proteins, *Pfmsp1* and *Pfmsp2* could be explained by balancing selection occurring as a result of different mechanisms of interaction with the host [42]. This distribution of families of *msp*-*1* and *msp*-*2* and their allelic variations were similar to that reported from other countries with meso- to high endemicity of malaria [43]. Genotyping procedure recommended in anti-malarial drug trials stipulates consecutive analysis of the three markers starting with *msp2* or *glurp*, and then *msp1* [23]. Based on allelic profile of each gene obtained in this study, parasitological outcome assessment could be more accurate when both markers *msp*-*1 and msp*-*2* are included in the genotyping of recurrent parasitaemias in anti-malarial drug trials, consistent with other studies [21]. The results of this study equally raise concern over the use of *glurp* genotyping in anti-malarial drug trials since this marker showed limited allelic families as compared to *msp*-*1* and *msp*-*2*. However, genotyping by SNPs and indels employing NGS could show more allelic families than identified by nPCR [44, 45].

### Genetic diversity for *msp*-*1* and *msp*-*2* allelic families

The present study reported higher numbers of alleles (43 alleles for both MSP-1 and MSP-2) than previously reported to be circulating in the study area and in the central region of Cameroon [11, 24]. A peculiarity of this study is that, RO33 was found to be monomorphic and the most predominant allele type of msp1 compared to the polymorphic K1 and MAD20 allelic families in agreement with previous studies where RO33 was found to be the most predominant allelic family for *msp1* locus [19, 35, 43]. However, the results of this study does not corroborate other studies in which RO33 was identified as the least predominant allelic variant type while MAD20 was the most predominant allelic family of the *msp1 l*ocus [16, 24]. This discrepancy could be attributed to the difference in the degree of transmission intensity. Another peculiarity of our study is that, the RO33 family of *msp*-1 did not show any polymorphism, with only 1 variant (155 bp) detected. This result differs from that of Gabon and West Uganda, where the Ro33 family was polymorphic with three and four allelic variants, respectively [8, 46], but was close to that in Senegal [47], and Brazil [48], showing the monomorphic nature of RO33 family of *msp*-*1*. The allelic variant K1 was the second highly distributed after RO33. This does not corroborate previous finding in the same region of Cameroon [24], but is consistent with most findings in areas of holoendemic, mesoendemic, and hyperendemic malaria, in which the allelic variant K1 was predominant [19]. The predominance of RO33 and K1 allelic family could be attributed to the balancing selection acting on these two allelic variants. The MAD20 allelic variant was the least predominant among the *Pfmsp1* allelic family in agreement with other studies conducted Africa including the Gambia, Nigeria, and Gabon [35]. This result does not corroborate with other studies reporting the predominance of MAD20 allelic variant over K-1 and RO33 allelic variants [13, 19]. The low distribution of MAD20 allelic variant could be partly explained by purifying selection acting on MAD20 allelic type as compared with RO33 and K1 allelic type. This could equally be attributed to a single nucleotide substitution in DNA template that hinders proper annealing with primers designed to amplify the MAD20 allelic type [49]. An association between the distribution of K1 allelic families with severe malaria has been investigated [43], while the RO33 allelic family has been frequently reported in asymptomatic malaria cases [16, 50]. The predominance of RO33 seems to be less harmful for the host since the presence of this allele type is related to reduced risk of clinical malaria [43, 51].

A significant correlation has been established between the genetic diversity of the *msp*-1 gene of *P. falciparum* and parasite density. Likewise an association has been observed between *msp*-*1* allele diversity and age group on one hand, and between *msp*-*1* allele diversity and gender among asymptomatic patients on the other hand [52].

The *msp*-*2* allelic families IC/3D7 and FC27 were almost of equal frequencies which is consistent with other findings [36]. In contrast, previous reports showed a significant predominance of FC27 over the 3D7/IC allelic family [16]. The frequencies of individual *msp2* genotypes were low with 88.9% occurring at a frequency ≤ 10%. However 2 (16.7%) genotypes, belonging to the FC27 and 1 (6.7%) belonging to the 3D7/IC allelic family were found at frequencies of above 10%. High genetic diversity and low allelic frequencies have been reported previously from other sites including Gabon [8], Uganda [22], Senegal [53], and Burkina Faso [54].

Diversity, expressed as expected heterozygote (*He*), ranged from 0.55 for *msp*-*1* to 0.96 for *msp*-*2* suggesting that the parasite population in Cameroon, exhibits intermediate to higher heterozygosity reflecting intermediate to high transmission pattern [14] consistent with the findings in Uganda, Congo and Zimbabwe, showing a high heterozygote ranges between 0.78 and 0.8 for *msp*-*2* [16, 55]. The correlation between the genetic variation of *P. falciparum* and malaria endemicity has been described [16, 46]. In areas with declining endemicity, it is reported that the number and diversity of alleles decrease with decreasing *P. falciparum* transmission [55]. The present study reveals that the *msp*-2 gene is highly polymorphic as compared with *msp* 1 gene. This observation is different in low transmission settings where high diversity has been recorded for *msp*-1 as compared with the *msp*-2 gene [13]. Hence, the high allelic diversity together with the low frequency of individual circulating alleles observed in the present study increase the discriminatory power of *msp*-1 and *msp*-2 to differentiate between recrudescence and re-infection. Thus, this study reinforce the importance for the genotyping of *P. falciparum* based on *msp*-1 and *msp*-2 in anti-malarial drug efficacy trials, to distinguish between re-infection as recrudescence and emphasize on the importance of implementing *msp*-*1* and *msp*-*2* genotyping in effective malaria management and malaria control strategies in Cameroon and in other endemic areas.

### Polyclonal infection expressed as MOI values

In this study, most patients with *P. falciparum* infections were infected with multiple genetically distinct parasite variants with high level of polyclonal infections observed among *msp*-2 (88.6%) and *msp*-1 (33.8%) positive isolates. Such a pattern of parasite structure is typical among *P. falciparum* populations in areas of high transmission, where more than 10 variants can be routinely detected in an individual [41], and selection among these variants in the host is likely to play an important role in parasite diversity. In contrast, in areas of low transmission, such as in Asia or in Latin America, patients may have infections with as few as a single variant [17]. No data on MOI was available in Cameroon before the introduction of ACT. This renders it difficult to draw a conclusive statement on the impact of ACT on MOI. Nevertheless, in this study, the polyclonal infection expressed as the MOI values were heterogeneous across the different loci, and the mean MOI was highest for *msp*-2 than *msp*-*1* in accordance with previous studies in neighbouring countries with high intensity of malaria transmission including Congo and Gabon [56]. Higher MOI in Cameroon can be the results of multiple infectious mosquito bites or transmission of genetically diverse sporozoite inoculum from a single mosquito bite. Genetically distinct malaria parasites in natural populations have an extremely high rate of genetic recombination during the sexual stages resulting in multiple strains being transmitted simultaneously [57]. Effective recombination of parasites and mutation occurring in several rounds of DNA replication cycles will likely continue to maintain this genetic diversity. During genetic recombination, novel combinations of alleles can be generated offering beneficial features to the parasite, as driven by positive selection enabling spread alleles through the population. Since the evolutionary selection of malaria occurs both within individual hosts and within populations, determining the number of strain in an infection might be an important indicator of transmission intensity [58]. Effective malaria control measures (ACT, distribution of ITNs) have successfully reduced malaria transmission in many hyperendemic regions of sub-Saharan Africa. After an intervention, the malaria parasite population structure and transmission rate in these regions is expected to become similar to the low transmission rates of the regions of Southeast Asia and South America. The declining malaria transmission, as a result of scaling up interventions, has been shown to affect the genetic diversity pattern and population structure of *P. falciparum* [18]. Therefore, the high MOI observed in this study reflects a high intensity of malaria transmission in Cameroon, despite several control strategies deployed at the health facilities and in the community. This is in agreement with previous findings that observed an increase of MOI with an increase in malaria endemicity and a low MOI for *msp*-1 and *msp* -*2* correlated with a low intensity of malaria transmission [17].

Therefore, regular molecular epidemiological surveys need to be performed in order to monitor the genetic diversity of *P. falciparum* populations in different regions of Cameroon, and then correlate parasite genotypes to the disease phenotypes [52, 59]. Previous studies reported a reduced risk of clinical malaria associated with polyclonal infections and high rate of severe malaria in individual harbouring mono-infections and very common genotypes [60]. This study showed an increase in the mean MOI according to parasite density, but not according to the gender of patients which is consistent with previous studies showing significantly high MOI in patients with moderate to high transmission [57]. In the present study, most of the positive samples were from children aged 0–5 years, and this limited range of age constraint examining the correlation between the MOI and age. However, previous studies showed a pattern of greater MOI in older individuals than younger individuals reflecting more previous exposure to infection [61] while conflicting findings, indicated decreased MOI with age [62]. Thus, determining the MOI in endemic areas is very important since it can be used to predict clinical outcome and target population with higher attention.

### Genotyping by gel electrophoresis and direct sequencing

In this study, the polymorphic surface antigens *msp2*, *glurp*, and *msp1* genes for 304 isolates were successfully amplified by primary and nested PCR and genotyped using agarose gel electrophoresis of which 127 *msp*-*1* and 297 *msp*-*2* gene fragments with single band were selected for further characterization by direct sequencing for identity and MSA purposes, but not to determine the entire parasite population. In this study, the lengths of the repeat units, whose number varies between the different allelic variants, was taken into account to set bin width of 20 bp for *msp*-*1* and *msp2* then 50 bp for *glurp* [14]. However, variations in bin width [22] and the different fragment sizing methods need to be standardized to facilitate the comparison of data for a particular marker between studies. Moreover, genotyping using agarose gel electrophoresis only groups alleles of similar size, but cannot distinguish the presence/absence of SNPs across the gene markers. Length polymorphic markers could introduce bias during the amplification process as this method is known to preferentially amplify shorter fragments [37, 63]. Although, genotyping method using gel electrophoresis may face some confounding factors including the variability in the electrophoretic migration of a given DNA fragment, this genotyping method could be very important where alternative method are not available and is widely used for *P. falciparum* genotyping [27, 28, 36, 64, 65]. Capillary electrophoresis-PCR (CE-PCR) was not used in this study, which could increase the allele resolution in an agarose gel by determining differences between 2 and 3 bp sizes among bands [14]. Beside, capillary electrophoresis is recognized to have higher resolution power than gel electrophoresis in the ability to distinguish between allelic variants of amplified fragments [20]. However, PCR artefacts are a challenge for this method and MOI determination is often underestimated not only with gel electrophoresis but also with capillary electrophoresis analysis especially when genotyping strategy do not consider separate nPCRs for each allelic family [21]. In contrast to previous studies, a separate nPCR followed by direct sequencing was performed in the present study enabling accurate determination of the fragments sizes for *msp1* and *msp2* allelic families as well as sequence motifs and nucleotide differences. The development of single-nucleotide polymorphism-based (SNP) genotyping techniques and next-generation sequencing (NGS), might provide highly diverse haplotype markers with sufficient resolution to detect minority population in a mixed infection [44, 45]. Thus, next-generation sequencing technologies and genome-wide characterization is an alternative strategy to accurately analyse polyclonal infections although complete haplotype characterization of multiclonal infections remains a challenge due to PCR artefacts and sequencing errors [66]. Therefore, in order to accurately study the competition and selection between variants in mixed malaria infection, new tools, more sensitive to detect minority populations and quantitative for relative parasite population sizes have being developed including DADA2, PASEC, HaplotypR, SeekDeep among others [45].

Another strategy could be the combination of sequencing method and efficient computational tool for an effective characterization of allelic variants. A good number of software packages are being developed to analyse genome-wide SNP data of field isolates for the estimation of the presence of multiple genotypes, especially minor allele in multiclonal infections [67]. However, DNA electrophoresis followed by direct sequencing should be used where alternative method are not yet available while waiting for the implementation of newly developed methods for allele genotyping, especially in the surveillance of malaria transmission. This would provide a guideline to policy makers to redefine the malaria control strategies.

### Sequence analysis of genetic polymorphism of *msp*-*1* and *msp*-*2*

This study showed highly diverse nature of *P. falciparum* isolates of Cameroon in respect to length and sequence motifs. Sequencing and gene alignment confirmed the identity *Pfmsp1* and *Pfmsp2* polymorphisms. Thus, when performing the gene alignment, high similarity was observed between the peptides of *Pfmsp1* and *Pfmsp2* in Cameroon and those of other regions in Africa. However, from all peptides analysed, the region of the alignment corresponding K1 polymorphism had the highest similarity among all the species in the *Pfmsp* clade included in this study ranging from 93% to 99% homology with previously described polymorphism in isolate from Kenya and Tanzania. The MAD20 peptide sequence polymorphism was the second most conserved with 83% to 100% homology between *P. falciparum* isolates in Cameroon and those of other regions of Africa as well as with those of other regions of the world. Thus, the development of a vaccine based on K1 and MAD20 allelic variant could likely be effective in providing immune protection against malaria in those regions in Africa, although it is not yet known to what extent the high allelic diversity within the K1-like and MAD20-like allelic types is of immunological significance [42].

However, previous analysis indicated more serological variation among the allelic sequences of the K1-like compared to the MAD20-like type [31], and more effort has been made to incorporate the repeat sequence variation of the K1-like alleles in recombinant antigens towards design of a multivalent vaccine [41, 49] and more than 500 different *msp*1 block 2 allelic sequences has been described, providing a reference for molecular epidemiological studies and potentially for design of a multi-allelic vaccine [42]. Sequencing and immunological characterization of other allelic variants such as MAD20 for *Pfmsp1*, alongside with 3D7/IC for *Pfmsp 2* should be conducted to obtain more useful information.

## Conclusion

In this first study conducted 5 years after the introduction of ACT, the genetic diversity of *P. falciparum* isolates was investigated among children aged 6 months to 6 years. The present study shows that field isolates of South West Region of Cameroon were found to be mainly polyclonal with high MOI and highly diverse in respect to both *msp*-*1* (block 2) and *msp*-*2* (central repeat region, block 3). These markers appear to be highly polymorphic with low allelic frequencies as compared to *glurp*. This observation reinforced the value of *msp*-*1* and *msp*-*2* markers of *P. falciparum* for PCR correction of treatment outcomes in classifying recurrent post-treatment *P. falciparum* episodes as recrudescence or new-infections in drug clinical trials. This study lays emphasis on the use of both *msp*-*1* and *msp*-*2* genes in monitoring the trend of malaria epidemiology and the use of MOI as an important indicator in the evaluation of malaria control interventions. The high MOI observed in this study is an indication that malaria transmission remains high in Cameroon despite a large distribution of ACT and calls for intensifying control interventions. Besides, the findings reveal that population structure of *P. falciparum* isolates is identical in Cameroon as shown by presence of common allelic composition and the high level of identity among allelic sequences from Cameroonian isolates and that of other areas in Africa and in the world and that *P. falciparum* population is a mixture of different strains. A vaccine developed with K1 and MAD20 of *Pfmsp1* allelic variant could be protective for Africa children, but this finding will require further genetic and immunological characterization. It is anticipated that clonal selection could not be uniform across the country considering the varied climate range observed in Cameroon moving from the northern region (with Sahel desert) to the south (with equatorial dense forest) corresponding to different malaria epidemiology. Therefore, it will be relevant to carry out studies on the genetic diversity of *P. falciparum* isolates from other regions of Cameroon with varied malaria epidemiology as well as longitudinal studies involving other malaria transmission related markers such as CSP to further understand the clonal fluctuations associated with transmission intensity.

## Data Availability

All data generated and/or analysed during this study are included in this published article (and its additional files) and are available from the corresponding author on reasonable request.

## Abbreviations

ACT: Artemisinin-based combination therapy
CDC: Cameroon Development Corporation
EDTA: Ethylene diamine tetraacetic acid
He: Expected heterozygosity
MOI: Multiplicity of infection
Pfmsp1: *Plasmodium falciparum* merozoite surface protein 1
Pfmsp2: *Plasmodium falciparum* merozoite surface protein 2
Glurp: Glutamate rich protein
CSP: Circumsporozoite protein
PCR: Polymerase chain reaction
TBE: Tris-borate EDTA
